# Grand Challenges in the Research of Fungal Interactions With Animals

**DOI:** 10.3389/ffunb.2020.602032

**Published:** 2020-10-30

**Authors:** Chengshu Wang

**Affiliations:** ^1^CAS Center for Excellence in Molecular Plant Sciences, Shanghai Institute of Plant Physiology and Ecology, Chinese Academy of Sciences (CAS), Shanghai, China; ^2^CAS Center for Excellence in Biotic Interactions, University of Chinese Academy of Sciences, Beijing, China

**Keywords:** host specificity, effector, immune evasion, antifungal immunity, chemical biology, community ecology

Different species of fungi and oomycetes play intimate pathogenic, symbiotic, physiological and ecological interactions with animals. Life threatening issues caused by fungi have been frequently reported not only to humans but also to other animals like amphibians and bats. For example, in addition to the invasive human diseases caused by the commensal fungi such as the species of *Cryptococcus, Candida, Aspergillus, Coccidioides*, and *Pneumocystis* genera, emerging threats have also been clinically reported from the infection of *Fusarium* and Mucormycetes fungi to the patients with a compromised immune system (Bongomin et al., 2017; Limon et al., 2017). The amphibian panzootic chytridiomycosis caused by the fungus *Batrachochytrium dendrobatidis* (Bd) has been causing the decline of about 500 amphibian species across continents over the past 50 years, a detrimental threat to ecosystem diversity and balance (Scheele et al., 2019). It is also ecologically problematic regarding the spread of the white-nose syndrome (WNS) disease caused by the psychrophilic fungus *Pseudogymnoascus destructans* (Pd) in bat populations (Puechmaille et al., 2011; Hoyt et al., 2020). It is technically challenging to investigate fungal infections in the populations of fish, shrimp and prawn which are frequently infected by fungi and Oomycetes, threatening the fishery and aquaculture industries to different levels (Gozlan et al., 2014). Otherwise, invertebrate pathogenic fungi have been well-investigated for their contributions to and functions in maintaining ecological balance and usages for biocontrol of agricultural pests (Li et al., 2015; Wang and Wang, 2017). Fundamentally, the conserved innate immune pathways, especially the antifungal Toll pathway, have been established by using the model organism *Drosophila melanogaster* as a genetically tractable system (Lemaitre et al., 1996; Ferrandon et al., 2007). However, overall, relative to the well-studied mechanisms (e.g., gene-for-gene theory) of fungi-plant interactions (Yan and Talbot, 2016; Fernandez and Orth, 2018), the knowledge obtained for fungi-animal interactions is still fragmentary due to, if not all, the unilateral research focuses on either fungal pathology or host immunology. Many challenges still exist both mechanistically and technically in the research and understanding of the dynamic and diverse types of the defense/antidefense and temporal/spatial interactions between fungi and animals.

## Challenges in Understanding the Mechanisms of Fungal Host-Specificity and Host-Preference

Except for the saprophytic fungal species, fungi evolved into different pathotypes to infect either plants, metazoans, or other fungal species (i.e., the mycoparasites). Comparative genomic studies revealed that ascomycete insect pathogenic fungi are more closely related to plant pathogens than to mammalian pathogenic fungi (Shang et al., 2016). On top of this, individual fungal species evolved with a varied capacity to infect different ranges of hosts. Similar to the incomplete understanding of the origin of species, it is biologically interesting but mechanistically intriguing to understand the formation of fungal host-specificity even between the closely related fungal species. It has been recognized that host-pathogen interactions are one of the major driving forces leading to the diversification and (co-)speciation of both pathogens and hosts, which have resulted in the formation of the fungal species with different host ranges or strains with preference for different host species (Hu et al., 2014; Mei et al., 2020). For example, different species of the insect pathogenic *Metarhizium* fungi evolved with different host ranges of which the generalist species like *M. brunneum, M. robertsii, M. anisopliae*, and *M. pingshaense* can infect hundreds of insect species whereas the specialists such as *M. acridum* is acridid-specific and *M. cicadae* is found to only infect cicadas (Hu et al., 2014; Mongkolsamrit et al., 2020). The species of zombie-ant fungi *Ophiocordyceps* spp. even evolved to the level of one-fungus for one-ant species (Wichadakul et al., 2015; Araujo and Hughes, 2019). In addition, it has been found that different isolates of *Beauveria bassiana* demonstrated a varied degree of pathogenicity against the same insect species, the feature of isolate host preference (Mei et al., 2020). Similar characteristics have been found for the field-collected strains of the nematode trapping fungus *Arthrobotrys oligospora* against *Caenorhabditis elegans* (Yang et al., 2020). This kind of host specificity and host preference of insect/nematode pathogens is of important biological implications; however, the underlying mechanisms are still poorly understood (Kirzinger and Stavrinides, 2012). In addition to the metabolic associations (Xu et al., 2016; Tong et al., 2020), divergent G-protein coupled receptors (GPCRs) have been revealed in association with fungal host-specificity (Shang et al., 2020). However, the host ligand molecule(s) recognized by individual fungal GPCR remains elusive. In contrast to the concept of the non-host resistance (NR) raised for plant hosts (Ayliffe and Sorensen, 2019), animal NR responses have not been investigated. It is tempting to speculate if NR is present in animal hosts there will be a connection between it and fungal host specificity. Much effort is still required to tackle the challenges in understanding the secrets involved in fungal selection of animal hosts to infect, kill and digest for nutrients.

## Challenges in Unveiling the Innate Effector Mechanisms Between Fungi and Animal Interactions

Remarkable similarities have been demonstrated in the innate immune defense systems across plants, mammals and insects (Nurnberger et al., 2004; Leulier and Lemaitre, 2008). However, relative to the gene-for-gene theory established for the microbe-plant interactions (Jones and Dangl, 2006; Jones et al., 2016), the concept of animal fungal effectors is still vague, and the innate effector mechanism has even been questioned for the interactions between fungi and animals since either the intimate apoplast structure is not present between fungal and animal cells (Lowe and Howlett, 2012; Cen et al., 2017), or the intracellular nucleotide binding-leucine-rich repeat immune receptors are largely missing in animals (Jones et al., 2016). During the studies of human and insect immunology, fungal pathogens have been largely treated as latent immunogenic factors, i.e., mainly the PAMP (pathogen-associated molecular pattern)-triggered immunities (PTIs) (Ferrandon et al., 2007; Briard et al., 2019). For example, different non-entomopathogenic fungi such as *A. fumigatus, A. nidulans, C. albicans, C. glabrata* and *Fusarium* fungi were used in the innate immunity studies of *D. melanogaster* by injection of live or dead fungal spores, which helped to establish the antifungal Toll pathway (Lemaitre et al., 1996; Ferrandon et al., 2007). Due to the common structure of fungal cell walls (Latgé et al., 2017), induction and activation of the *Drosophila* Toll pathway by non-pathogenic fungi are not surprising. In nature, however, entomopathogenic fungi infect and kill insects through the process of cuticle penetration and host hemocoel colonization along with cell wall structure remodeling, phenotype switch and inhibition of host immune defenses (Wang and Wang, 2017). Thus, caution has to be taken during the investigation of the interactions between fungi and animals.

Apart from PTIs, accumulated evidence has indicated that the innate effector mechanisms, i.e., the effector-trigged immunities (ETIs), are similarly present between microbe and animal interactions (Lopes Fischer et al., 2020). For example, the plant-pathogen effector-like proteins such as the small secreted cysteine-rich proteins (SSCPs) are similarly present in animal pathogenic fungi (Shang et al., 2016). Functional studies have revealed that the LysM effectors of the plant pathogenic fungi are also present in the entomopathogens such as *B. bassiana* with the chitin-binding activities to evade insect immunities (Cen et al., 2017). By using the *Drosophila* model, the serine protease PR1 of entomopathogenic fungi has been revealed as a “danger molecule,” which can be detected by the host protease Persephone to boost the activation of the antifungal Toll pathway (Gottar et al., 2006; Issa et al., 2018). The metalloprotease of the M35 family of *M. robertsii* can directly cleave and inactivate the immune enzyme prophenoloxidases in insects to downregulate host immune defenses (Huang et al., 2020). Besides the cell wall PAMPs, a SSCP-like effector Sel1 of *C. albicans* could function as a PAMP molecule by interacting with the Toll-like receptors TLR2 and TLR4 to activate the NF-κB and MAPK (mitogen-activated protein kinase) pathways in mice (Wang et al., 2019). It has also been found that the small RNA encoded by *B. bassiana* could be deployed by the fungus to target and downregulate the expression of the Toll-pathway genes in mosquitos (Cui et al., 2019). Overall, the ETI mechanism of fungi-animal interactions is highly expected but limitedly understood what requires extensive research efforts by using different systems in the future.

## Challenges in Revealing the Small-Molecule-Mediated Interactions Between Fungi and Animals

Metabolomic studies have shown that differential accumulation of hundreds to thousands of metabolites could be detected from either fungi or animal hosts during fungi and animal interactions (Xu et al., 2015; Gonçalves et al., 2017; Kuo et al., 2020). Thus, in addition to the identification of effector-like proteins, small molecules produced by fungi and animals may also be involved in cross-kingdom interactions. Indeed, different studies have revealed that small molecules, especially the secondary metabolites, are virulence factors of animal pathogenic fungi. For example, the virulence factor glitoxin produced by *A. fumigatus* can target host integrins to induce anoikis (a type of cell apoptosis) on lung epithelial cells (Haun et al., 2018). Deletion of the compound biosynthetic genes and insect bioassays revealed that production of the secondary metabolites is required for fungal virulence against insects such as the cyclopeptides destruxins produced by *Metarhizium* species (Wang et al., 2012), beauvericin (Xu et al., 2008), and beauverolide/beauveriolide (Yin et al., 2020) produced by *B. bassiana* as well as the immunosuppression drug cyclosporine produced by the facultative beetle pathogen *Tolypocladium inflatum* (Yang et al., 2018). The studies have shown that these molecules could directly or indirectly subvert host immune defense systems. Overall, chemical biology studies of animal-pathogenic fungi may shed new light on the small-molecule-mediated interactions between fungi and animals. However, there are both technical and scientific challenges in identifying the virulence-related metabolites (VRMs) produced by fungi and the VRM targeted protein/receptor(s) of animal hosts.

## Challenges in Investigating Fungi-Animal Interactions in Nature

Animal mycosis occurs in different patterns in the fields like panzootic, epizootic and enzootic. The devastating panzootics of the bat WNS disease and amphibian chytridiomycosis are threating not only the safeties of the bat and frog populations but also the diversity and balance of different ecosystems. On the one hand, evolutionary, comparative and or population genomic studies have helped answer the regional origin, transmission, and pathogeography of the fungal pathogens (Puechmaille et al., 2011; Drees et al., 2017; O'hanlon et al., 2018; Scheele et al., 2019; Hoyt et al., 2020). On the other hand, however, very little is known regarding the factor(s) and mechanisms involved in determining the fate and consequence of the Bd and Pd infections of their hosts in nature. To meet these challenges, in addition to the urgent needs to set up the systems and models for laboratory mechanistic studies, fungal community ecology investigations of the Bd and Pd interactions with other microbes are necessarily required to help find if there is any practical way to block disease transmission and spread in host populations. Indeed, the studies have found that skin (co-habiting) microbiota on frogs could prevent morbidity and mortality caused by chytrid Bd (Harris et al., 2009; Mckenzie et al., 2012), and the bat cutaneous bacteria with antifungal activities had potential to protect bats from Pd infection (Grisnik et al., 2020).

Taken together with the pathogenic bacteria and viruses, invertebrate pathogenic fungi play important roles in maintaining host population density in different ecosystems (Wang and Wang, 2017). The ecology of invertebrate diseases has been studied for a long time, and has revealed the transmission, persistence, biotic/abiotic factor influence, and host density-dependent prevalence of invertebrate fungal pathogens (Hajek and Meyling, 2017). Depending on host population density, both epizootic and enzootic prevalence of fungal diseases can occur in invertebrate populations in the fields. Interestingly, co-epizootic of entomopathogenic fungi has also been found that different pathogens partitioned the habitat to attack the same insect host (Clifton et al., 2019). Different from the detrimental environmental threats caused by amphibian and bat pathogenic fungi, insect and nematode pathogens have long been used as environmentally friendly biocontrol agents (Wang and Feng, 2014). Thus, except for the similar requirement of the community ecology studies of invertebrate pathogens, environmental effects including biosafety of the biocontrol applications of entomopathogenic and nematophagous fungi require further investigations. A recent long-term monitoring and population genomic study of *B. bassiana* has revealed that the released strains to control pine caterpillars *Dendrolimus punctatus* could persist in the fields for a long time (> 20 years as being surveyed) but with low recovery rates and the ability to infect non-target insects, however, which demonstrated an enzootic pattern like local strains. In addition, in contrast to the arms-race model between pathogen and plant interactions (Moller and Stukenbrock, 2017), a trench-warfare scenario was suggested between the interactions of *B. bassiana* and insects in the field (Mei et al., 2020). Regarding the diverse fungi-animal interaction systems, more efforts are still required to meet the challenges in understanding the temporal and spatial interactions between fungi and animals in the fields.

## Conclusions

Both animals and animal pathogenic fungi are taxonomically diverse, and different strategies have evolved from both sides to maximize their own adaptive fitnesses, survivals and reproductions. The obtained knowledge has helped us to understand the common or unique interactive mechanisms adopted by either side. However, extensive research efforts are still required by using different models and systems to facilitate the understanding of the nature of fungal host specificity, effector mechanism and chemical biology of molecular interactions, and temporal/spatial patterns of ecological interactions in the fields. In addition, it is also mysterious regarding the mechanisms involved in fungal control of host animal behaviors (Shang et al., 2015; Lovett et al., 2020), and host (especially the social insects) social immune responses against fungal infections (Roy et al., 2006; Konrad et al., 2012), which also raise challenges for future investigations.

## Author Contributions

The author confirms being the sole contributor of this work and has approved it for publication.

## Conflict of Interest

The author declares that the research was conducted in the absence of any commercial or financial relationships that could be construed as a potential conflict of interest.
